# Medical News

**Published:** 1855-06

**Authors:** 


### MEDICAL NEWS.

The vacancy in the Chair of Surgery of the University of Pennsylvania, caused by the resignation of Prof. Gibson, has been filled by the election of Dr. Henry H. Smith, of Philadelphia.

Dr. Smith graduated in the department of Arts of the University of Pennsylvania, in the year 1834, and in the Medical Department of the same University in the year 1837. After being resident physician in the Pennsylvania Hospital for two years, Dr. S. went to Europe, where he spent 18 months. Upon his return, he lectured for several years in the Philadelphia Medical Institute. He was appointed Assistant Lecturer on Demonstrative Surgery in the University of Pennsylvania in the year 1845, and Consulting Surgeon to St. John's Hospital in 1849. In the year 1854 he was elected Consulting Surgeon and Lecturer on Clinical Surgery in the Philadelphia Hospital, Blockley.

In 1840, Dr. Smith translated Civiale on Stone and Gravel. In 1843, he published a work on Minor Surgery, which has since passed through three editions; and in 1844, an Anatomical Atlas. Edited the United States Dissector in 1846; and, in 1852, published his large work on Operative Surgery, now in its second edition.

Dr. Smith has recently sailed for Europe, to procure new material for the illustration of his course.

---

In a letter received from Dr. Ruschenberger, of the U. S. Navy, now attached to the flag ship Independence, is related the following instance of lofty tumbling.

"We had an odd accident. A seaman, off Cape Horn, in a gale of wind, was standing, on a level with the cap, in the weather main topmast rigging, to guide the main top-gallant yard in its descent from aloft. A heavy roll brought the yard against him and flung him off, head downwards. As he fell the main topmast back stay was embraced betwixt his body and left arm. When a little above the shearpoll a rope caught his feet and turned him over, so that he alighted on his feet in the main chains. He was brought in upon deck unhurt. He said he tried to seize hold of the back stay, but came down so —— fast he could not hold on. On being questioned he said he was not hurt, but felt ' considerably shook up.'

I remember a fat boy, who fell asleep in the fore-top of the U. S. S. Peacock, and rolled out. He fell on deck upon his side; his contusion was so slight he was not excused from duty a single day. He fell about 40 feet, but the spars of the Independence are those of a ship of the line, and her main cap is 100 feet above the deck.

---

*Abstract of Meteorological Observations for April,* 1855, *made at Philadelphia, Pa. Latitude* 39° 57′ 28″ *N., Longitude* 75° 10′ 40″ *W. from Greenwich.* By Prof. James A. Kirkpatrick.

| 1855. April. | Barometer. Daily Mean | Barometer. Mean Daily Range. | Thermom. Daily Mean | Thermom. Mean Daily Range | Dew Point 2 P.M. | Force of Vapor 2 P.M. | Rel. Humid. 2 P.M. | Rain and melted Snow. | Prevailing Winds. | Remarks. |
|---|---|---|---|---|---|---|---|---|---|---|
| | Inches. | Inches. | Deg. | Deg. | Deg . | Inches. | Hunds. | Inch. | Points. | |
| 1 | 29.355 | .369 | 43.7 | 11.2 | 27.3 | .123 | .31 | | NW. | M. cl'dy; aft. & ev. clear. *Bar.* |
| 2 | 29.602 | .247 | 34.0 | 12.3 | 21.3 | .077 | .32 | | W. | Cl'r. *Th. l'st* 22°. [*l'st* 29.234 |
| 3 | 29.963 | .361 | 37.7 | 3.7 | 33.3 | .182 | .60 | | NW. | Clear. |
| 4 | 30.116 | .153 | 44.3 | 6.7 | 24.7 | .097 | .22 | | Var.) | Aft. cloudy; m. and ev. clear. |
| 5 | 29.816 | .266 | 43.8 | 6.8 | 41.7 | .265 | .91 | 0 922 | NE. | Cloudy; ev. fog. *Rain* all day. |
| 6 | 29.562 | .254 | 52.0 | 8.2 | 36.7 | .229 | .44 | 0.255 | WNW. | Cloudy; ev. and night *Rain.* |
| 7 | 29.700 | .139 | 46.0 | 6.0 | 32.2 | .172 | .43 | | NW. | Clear. |
| 8 | 29.771 | .071 | 47.3 | 4.0 | 35.7 | .216 | .43 | | SW. | M. fog; aft. and ev. clear. |
| 9 | 29.805 | .034 | 50.0 | 6.7 | 46.0 | .321 | .80 | 0.072 | SW. | Cloudy; m. and ev. *Rain.* |
| 10 | 29.785 | .084 | 49.3 | 1.3 | 48.3 | .349 | .86 | 0.254 | (Var.) | Cloudy; drizzling rain all day |
| 11 | 29.791 | .080 | 40.3 | 9.0 | 33.3 | .186 | .82 | | NW. | M. cl'r; a. & ev. cl'dy. 1 to 2 P.M |
| 12 | 29.966 | .171 | 49.3 | 9.0 | 25.7 | .107 | .24 | | WNW. | M. cl'dy; a. & ev. cl'r. [*Snow.* |
| 13 | 29.980 | .053 | 52.5 | 3.2 | 34.3 | .203 | .39 | | (Var.) | Cloudy. |
| 14 | 29.773 | .207 | 55.7 | 3.2 | 46.0 | .339 | .65 | | (Var.) | Cloudy. |
| 15 | 29.789 | .079 | 50.7 | 7.0 | 47.7 | .335 | .93 | 0.331 | NE. | *Rain* till 6 P.M.; ev. clear. |
| 16 | 30.045 | .250 | 55.3 | 9.7 | 37.0 | .244 | .40 | | ( Var.) | Clear. [2¼ P.M. |
| 17 | 30.016 | .079 | 54.0 | 3.0 | 41.3 | .283 | .54 | 0.006 | SW. | Cloudy. *Shower* from 1¾ to |
| 18 | 29.794 | .222 | 64.0 | 8.0 | 53.3 | .490 | .63 | | ENE. | M. cloudy; aft. and ev. clear |
| 19 | 29.648 | .146 | 73.3 | 9.3 | 48.7 | .517 | .42 | | NNE. | Cl'dy. *Th. highest,* 4 P.M. 87° |
| 20 | 29.650 | .098 | 57.0 | 16.3 | 46.0 | .339 | .65 | 0.308 | (Var.) | M. and aft. cloudy; ev. clear; from 3 to 4 P.M *rain, th'r, l'ng.* |
| 21 | 30.073 | .423 | 55.3 | 5.0 | 37.0 | .244 | .40 | | NW. | Clear. |
| 22 | 29.919 | .154 | 57.3 | 4.7 | 47.7 | .372 | .63 | | SW. | M. and aft. cloudy; ev. clear. |
| 23 | 30.039 | .120 | 60.0 | 7.3 | 40.0 | .290 | .42 | | ENE. | Clear. |
| 24 | 29.959 | .118 | 61.8 | 8.5 | 55.3 | .510 | .68 | | SW. | Cl'y; m. fog. [8 to 9 P.M. *th. ltg.* |
| 25 | 29.869 | .151 | 74.0 | 12.5 | 48.7 | .517 | .42 | | (Var.) | Cl'dy. *Th. hig'st* at 3 P.M. 87°. |
| 26 | 29.811 | .136 | 56.7 | 17.3 | 48.7 | .365 | .76 | | (Var.) | Cloudy; a few drops of rain |
| 27 | 30.055 | .244 | 56.0 | 4.0 | 32.7 | ·189 | .33 | | NW. | Clear. [at 5 P.M. |
| 28 | 30.113 | .058 | 55.3 | 0.6 | 32.7 | .189 | .33 | | W. | M. & ev. clear; aft. cl'dy. *Bar.* |
| 29 | 29.982 | .132 | 56.2 | 3.8 | 43.3 | .335 | .47 | | NE. | Cloudy. [*highest* 30.179. |
| 30 | 29.925 | .057 | 53.5 | 5.0 | 46.0 | .339 | .65 | | NE. | Cloudy. |
| Means for April, 1855 | 29.856 | .165 | 52.9 | 7.1 | 39.8 | .281 | .54 | 2.148 | N. 52° W., 39–100. | |
| 4 yrs | 29.808 | .193 | 50.6 | 7.2 | 39.1 | | | 5.153 | N. 53½° W. 33–100. | |

The Monthly Range of the Barometer was 0.945 of an inch., and of the Thermometer 65°.

